# Transcriptional and metabolite analysis reveal a shift in direct and indirect defences in response to spider-mite infestation in cucumber (*Cucumis sativus*)

**DOI:** 10.1007/s11103-020-01005-y

**Published:** 2020-04-18

**Authors:** Jun He, Harro J. Bouwmeester, Marcel Dicke, Iris F. Kappers

**Affiliations:** 1 Laboratory of Plant Physiology, Wageningen University and Research, Wageningen, The Netherlands; 2 Swammerdam Institute for Life Sciences, University of Amsterdam, Amsterdam, The Netherlands; 3 Laboratory of Entomology, Wageningen University and Research, Droevendaalsesteeg 1, 6708 PB, Wageningen, The Netherlands

**Keywords:** *Cucumis sativus*, Herbivores, Secondary metabolism, Transcriptome, Two-spotted spider mites, Volatile organic compounds

## Abstract

**Key message:**

Cucumber plants adapt their transcriptome and metabolome as result of spider mite infestation with opposite consequences for direct and indirect defences in two genotypes.

**Abstract:**

Plants respond to arthropod attack with the rearrangement of their transcriptome which lead to subsequent phenotypic changes in the plants’ metabolome. Here, we analysed transcriptomic and metabolite responses of two cucumber (*Cucumis sativus*) genotypes to chelicerate spider mites (*Tetranychus urticae*) during the first 3 days of infestation. Genes associated with the metabolism of jasmonates, phenylpropanoids, terpenoids and l-phenylalanine were most strongly upregulated. Also, genes involved in the biosynthesis of precursors for indirect defence-related terpenoids were upregulated while those involved in the biosynthesis of direct defence-related cucurbitacin C were downregulated. Consistent with the observed transcriptional changes, terpenoid emission increased and cucurbitacin C content decreased during early spider-mite herbivory. To further study the regulatory network that underlies induced defence to spider mites, differentially expressed genes that encode transcription factors (TFs) were analysed. Correlation analysis of the expression of TF genes with metabolism-associated genes resulted in putative identification of regulators of herbivore-induced terpenoid, green-leaf volatiles and cucurbitacin biosynthesis. Our data provide a global image of the transcriptional changes in cucumber leaves in response to spider-mite herbivory and that of metabolites that are potentially involved in the regulation of induced direct and indirect defences against spider-mite herbivory.

**Electronic supplementary material:**

The online version of this article (10.1007/s11103-020-01005-y) contains supplementary material, which is available to authorized users.

## Introduction

Plants have two types of defence strategies that affect herbivores either in a direct or an indirect way and both strategies can be constitutively present or be induced upon herbivory (Mithofer and Boland 2012). Physical and chemical barriers such as plant hairs, latex and leaf toughness can hinder herbivores to access the foliage. Specialized metabolites—constitutively present or synthesized upon attack—may deter herbivores from feeding or reduce herbivore performance and include a wide range of different biochemical classes (Mithofer and Boland 2012; Santamaria et al. 2013). In addition to changes in endogenous metabolites affecting the level of direct defence, volatile metabolites emitted from plants in response to herbivore attack can serve as cues to attract natural enemies of the herbivores (Dicke and Sabelis 1988; Turlings and Erb 2018).

Re-configuration of the metabolome, including endogenous compounds and emitted volatiles, results from prior re-configuration of the transcriptome in which multiple phytohormones and transcription factors (TFs) interplay to regulate the complex networks that up-regulate or suppress defence-related genes (Ozawa et al. 2000; Wu and Baldwin 2010). Plants perceive herbivory attack via damage- or herbivore-associated molecular patterns (Mithofer and Boland 2008) and upon recognition, phytohormones modulate the response to different attackers, although the exact mechanisms are unclear (Lazebnik et al. 2014). In general, jasmonic acid (JA) has been identified as the essential regulator of plant responses to various chewing herbivores, while salicylic acid (SA), ethylene and to a lesser extent other phytohormones interplay with JA to fine-tune the response to a specific plant–herbivore interaction (Broekgaarden et al. 2018). Subsequently, hormonal signalling is translated into activation or repression of gene expression. This transcriptional regulation usually depends on the transcription and translation of TFs (Wu and Baldwin 2010), although other factors such as chromatin-remodelling may also be involved (Berr et al. 2012). Different TF families, including MYB, bHLH, WRKY, AP2/ERF, NAC and bZIP play a role in plant defence (Seo and Choi 2015) and multiple of such TFs have been related to changes in the transcription of metabolism-associated genes, finally resulting in an altered metabolic profile of the infested plant (Wu and Baldwin 2010).

While chewing herbivores bite off and ingest large parts of plant tissue and sap-sucking herbivores hardly make visible mechanical damage during early infestation, chelicerate spider mites insert their needle-like stylets in between epidermal cells into single mesophyll cells and feed on the cell contents (Bensoussan et al. 2016), resulting in chlorotic spots and loss of photosynthetic capacity (Park and Lee 2002). JA and its derivatives have been implicated as key components of the defence response to spider mites in lima bean (Dicke et al. 1999), tomato (Ament et al. 2004; Martel et al. 2015; Schimmel et al. 2018), Arabidopsis (Zhurov et al. 2014) and in cereals including maize and barley (Bui et al. 2018).

Two-spotted spider-mites (TSSM, *Tetranychus urticae*) are cosmopolitan herbivores feeding on plants from more than 140 plant families (Migeon et al. 2010) including cucumber (Mercke et al. 2004; Kappers et al. 2010). Multiple studies indicate the importance of constitutive defences which confer resistance towards TSSM such as the presence of (glandular) trichomes (Glas et al. 2012) and specific metabolites such as acyl sugars in wild tomato relatives (Dias et al. 2013), cucurbitacins in cucumber (Balkema-Boomstra et al. 2003), indolic glucosinolates in Arabidopsis (Zhurov et al. 2014) and benzoxazinoids in maize (Bui et al. 2018). These specific metabolites were often found to be increased upon TSSM feeding including indole glucosinolates in Arabidopsis (Zhurov et al. 2014), flavonoid metabolites in citrus (Agut et al. 2014) and the bitter triterpenoid cucurbitacin C in cucumber (Agrawal et al. 1999). In addition, TSSM herbivory results in the emission of volatile organic compounds of which multiple have been identified to be attractive to natural enemies of TSSM, including methyl salicylate (MeSA) (De Boer and Dicke 2004), green leaf volatiles (GLV) and several terpenoids (Kappers et al. 2011).

Elucidating the expression of genes upon herbivory in plants and their mode of regulation is a prerequisite to gain a better understanding of the processes involved in plant–herbivore interactions. Using mutants in which JA biosynthesis or signalling is disrupted showed that a subset of the induced genes as result of TSSM infestation, is involved in JA signalling and in the biosynthesis of specialized metabolites (Zhurov et al. 2014). Comparison between TSSM-induced DEGs in Arabidopsis and tomato revealed a conserved role for genes involved in JA signalling, and the biosynthesis of phenylpropanoids, flavonoids and terpenoids in response to this herbivore while specifically in tomato gene sets related to anabolism were suppressed suggesting a possible shift from growth to defence (Martel et al. 2015).

Cucumber is one of most important vegetable crops in the world only preceded by tomato, onion, and cabbage and is of major economic and nutritional importance (https://faostat.fao.org). In the last decade more genomic resources became available for cucumber including de novo sequenced genomes, a re-sequenced core collection of germplasm, and multiple global expression datasets (Huang et al. 2009; Qi et al. 2013; Wang et al. 2018). Upon TSSM infestation, net photosynthetic rate and total chlorophyll content in cucumber can decrease up to 95% and 80%, respectively (Park and Lee 2002). TSSM infestation of cucumber leaves results in strong induction of volatile emission including (*Z*)-3-hexenyl acetate and various terpenoids such as (*E*)-4,8-dimethyl-1,3,7-nonatriene (DMNT), (*E*)-β-ocimene and (*E,E*)-α-farnesene (Takabayashi et al. 1994; Mercke et al. 2004). Moreover, volatile blends induced by TSSM or JA treatment attract the predator *Phytoseiulus persimilis* (Kappers et al. 2010), implying a role of the JA-mediated signalling pathway in the tritrophic interaction between cucumber, TSSM and predatory mites. In addition, resistance to TSSM could be linked to constitutive levels of cucurbitacin C (Balkema-Boomstra et al. 2003). Cucurbitacins occur widely in the Cucurbitaceae and are toxic to many organisms, including TSSM (Agrawal et al. 1999; Balkema-Boomstra et al. 2003). Cucurbitacin C is the only cucurbitacin identified in *Cucumis sativus* (Balkema-Boomstra et al. 2003) and has been reported to increase in the cotyledons of a bitter cucumber genotype upon TSSM feeding (Agrawal et al. 1999).

Previously, we identified a TERPENE SYNTHASE (TPS) and a LIPOXYGENASE (LOX), that are both involved in the biosynthesis of volatiles present in the blend of TSSM-infested cucumber plants (Mercke et al. 2004). To obtain a more detailed understanding of the genes and pathways involved in induced responses to TSSM feeding in cucumber, we performed time-course transcriptome profiling of two cucumber varieties upon infestation with TSSM. Both genotypes differ in the presence of cucurbitacin C and were found to differ in their TSSM susceptibility. By analysis of the transcriptional changes we aimed to study which signal transduction and biochemical pathways are affected during the first 3 days of TSSM infestation with an emphasis on genes and transcription factors associated with metabolic reconfiguration.

## Material and methods

### Plants and mites

Seeds of cucumber (*C. sativus*) accessions with bitter (Chinese long 9930, CL) or non-bitter foliage (Corona, CO) were germinated in potting soil and plants were cultivated in a greenhouse compartment (16 h photoperiod (22 ± 2 °C), 8 h night period (18 ± 2 °C). Two-spotted spider mites (*T. urticae*) and predatory mites (*P. persimilis*) were originally obtained from Koppert Biological Systems. TSSM were mass-reared in the laboratory on Lima bean (*Phaseolus vulgaris*) plants for many generations. To obtain females of the same age, 40 TSSM were transferred to a fresh plant for oviposition and subsequently removed after 24 h. Hatched female mites were considered to be of the same age (synchronized rearing). Predatory mites were maintained on TSSM-infested leaves for 3–4 days after delivery prior to experiments.

### TSSM performance

To compare TSSM oviposition performance on both cucumber accessions, synchronized female mites were transferred to the abaxial side of leaf discs (6 cm diameter) placed on water agar (0.5%) in a Petri dish. Leaf discs were incubated under dim light (to prevent mites to abscond) at 22 ± 1 °C for 7 days and the number of eggs was counted. For each accession 10 leaf discs with 2 female mites per disc were used. The experiment was repeated three times. Differences between genotypes were tested for significance using ANOVA after Levene’s test for equal variance.

### TSSM infestation

The abaxial surface of the first fully expanded leaf of 4-week-old plants was infested with 50 adult TSSM. Leaves were collected at different time points after the onset of infestation, ranging from 15 min to 3 days, and flash frozen in liquid nitrogen. To exclude differences in response due to gating in circadian rhythms, leaves were harvested between 13.00 and 14.00. Non-infested plants were used as control. Three biological replicates each containing leaves of two plants were generated for each time point and treatment. Gene transcripts and metabolites were all analysed from the same samples. To estimate damage inflicted by TSSM as percentage of the total leaf area, pictures of the adaxial surface were taken just prior to harvest, analysed using Image J (https://imagej.nih.gov) and tested for significance using ANOVA after Levene’s test for equal variance. For volatile analysis, separate plants were used.

### Predatory mite preferences

The volatile blends of TSSM-infested CO and CL plants were tested for their relative attractiveness towards adult female *P. persimilis* mites using a Y-shaped olfactometer as previously described (Kappers et al. 2010). Attractiveness was determined after one, two and 3 days of infestation using 20 mites per experiment. Experiments were repeated six times for each combination and tested for significance using chi-square tests.

### RNA extraction, qRT-PCR, cDNA library preparation and Illumina sequencing

Total RNA was extracted from leaf samples collected just prior to infestation, 15, 30, 60, 120, 180, 240 min, and 24, 48, 72 h after infestation, using the RNeasy Plant Mini Kit (Qiagen, Hilden, Germany) and DNaseI digested (New England BioLabs, USA) according to the manufacturer’s protocols. For quantitative RT-PCR, one µg of high-quality DNA-free RNA was reverse transcribed using the iScript cDNA syntheses kit (BioRad, USA). Gene specific primers (Supplemental Table S1) were designed based on sequences obtained by BLAST search in the cucumber genome database (www.cucurbitgenomics.org; v2.0). qRT-PCR analyses were performed using the SYBR Green Supermix Reagent (BioRad, USA) and the following PCR program: 3 min at 95 °C; 40 cycles of 10 s at 95 °C and 45 s at 57.5 °C. Dissociation curves were checked for the absence of non-specific PCR amplifications. Threshold cycle (Ct) values were normalized for differences in cDNA synthesis using *ACTIN* (Csa6M484600) using the $2^{{ - \Delta \Delta C_{{\text{t}}} }}$ method. Log_2_-transformed expression ratios were calculated for each experimental condition. Based on qRT-PCR results, we selected samples for RNA-seq. mRNA was enriched from total RNA and cDNA libraries were synthesized using random hexamer primers and reverse transcriptase according to manufacturing’s protocols (Invitrogen, USA). Libraries were sequenced by Illumina HiSeq™ (Illumina, San Diego CA, USA). Raw reads were processed using Perl (www.perl.org/) scripts to remove reads containing adapter or more than 10% unknown bases and low-quality reads. GC content and sequence duplication level of the clean data were calculated. Sequence saturation was measured by correlating reads number and detected genes. Downstream analyses were based on the high-quality clean data. These reads were mapped to the public cucumber genome (Huang et al. 2009, version 2) and annotation database of accession ‘Chinese long 9930’ using SOAPaligner/soap2 (https://soap.genomics.org.cn/soapaligner.html). Furthermore, reads were mapped to the genome sequences of TSSM (Grbic et al. 2011) using TopHat (Trapnell et al. 2012).

### RNA-seq data analysis

Reads Per Kilobase of transcript per Million mapped reads (RPKM) were used to calculate gene expression levels using the longest transcript of a given gene if there were more than single transcripts for that gene. DEGs were identified by comparing sequenced libraries from different time points after infestation with those of non-infested plants for accession CL and CO separately using a False Discovery Rate (FDR) of 0.01 and Log_2_ (Treated/Control) fold-change > 1 or < − 1 as thresholds. The list of DEGs responsive to downy mildew was obtained from Adhikari et al. (2012). Expression data of cucumber organs and TSSM-induced DEGs in tomato leaves are from published datasets (Li et al. 2011; Martel et al. 2015).

Genemath (www.applied-maths.com) was used for cluster analysis of DEGs using Log_2_-transformed and mean-centred expression data. Pairwise distances were calculated based on Pearson correlation and UPGMA (no weighted Pair Group Method with Arithmetic Mean) was used to summarize the distance between clusters. Detected cucumber genes were mapped to the general Gene Ontology (GO) (www.geneontology.org) database and enrichment analysis was performed using agriGO (www.bioinfo.cau.edu.cn>agriGO) based on GO terms as well as on the pathway-related KEGG database (www.genome.jp/kegg/kegg1.html). GO-terms with a Hochberg FDR corrected hypergeometric tests *P* < 0.05 were considered.

### Identification of TF genes in the cucumber genome

Sequences were aligned to online databases including Genbank non-redundant protein sequences database and InterPro protein signature databases (www.ebi.ac.uk/interpro/) to identify genes encoding proteins with specific domains for different TF families. TFs in the collection of TSSM-induced DEGs were identified as responsive TF genes and selected for further analysis.

### Enrichment analysis of motifs

The 2 kb promoter regions of the up-regulated and down-regulated genes were extracted from the published cucumber genome (Huang et al. (2009), version 2) and motif enrichment in each group of promoters was performed by aligning promoter sequences to the database of DNA-binding specificities of plant transcription factors in Arabidopsis (Franco-Zorrilla et al. 2014). Adjusted *P* values were calculated using Wilcoxon rank-sum test and Bonferroni correction. The selected promoter sequences were searched using PlantCARE (https://sphinx.rug.ac.be:8080/PlantCARE/).

### Volatile analysis

Headspace of TSSM-infested or non-infested plants was collected on Tenax (20/35-mesh, Alltech) liners and subsequently analysed on a Thermal Desorber (TD100-xr, Marker, UK) connected to a GC-quadrupole time of flight mass spectrometer (QToF-MS, Agilent Technologies, USA) as described by Zhang et al. (2020). Individual compounds were semi-quantified by calculation of the peak area under the curve and identified by comparison of mass spectra to those of authentic standards. For each time point and accession, five plants were analysed. Differences in emission between accessions for different biochemical groups of volatiles on each consecutive day were tested using Student’s T-test.

### Cucurbitacin C quantification

Leaf samples of non-infested plants, and those infested with TSSM for 1, 2 or 3 days were analysed for cucurbitacin C. Hereto, 100 mg leaf material was extracted with 300 mL of MeOH (0.1% w/v formic acid), sonicated for 30 min, and centrifuged at 21,000* g* for 10 min. LC–MS profiling of the crude supernatant was performed using Synapt QToF LCMS based on De Vos et al. (2007). Mass chromatograms were processed using the MetAlign software package (www.wur.nl/nl/show-6/MetAlign.htm). The relative peak height of the representative mass [M+H]^+^ 560.33492 for cucurbitacin C was extracted at the retention time previously recorded for a pure standard.

## Results

### Accessions differ in TSSM-induced chlorotic spots, volatile emission and attraction of predatory mites

**Fig. 1:**
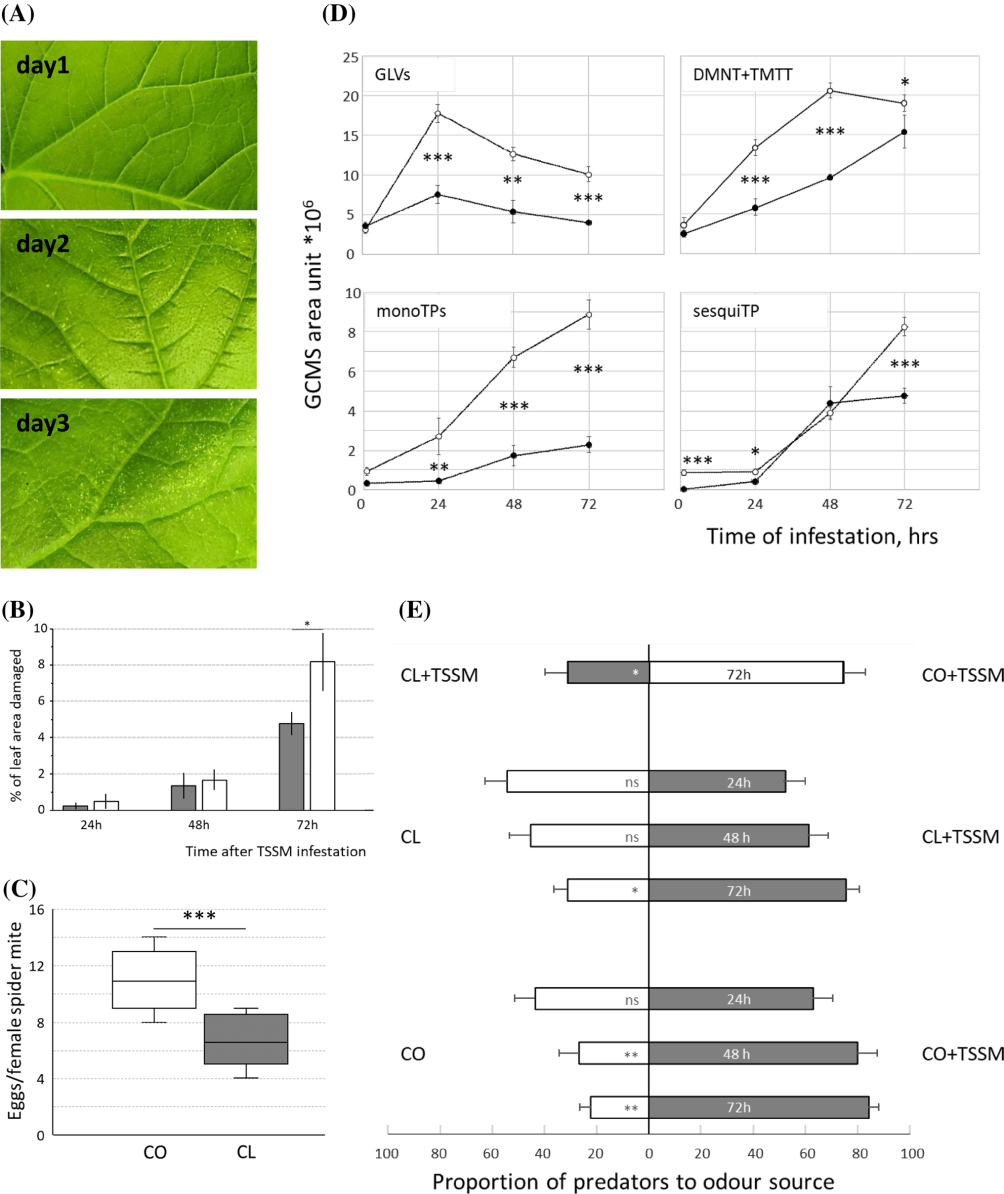
Defence responses in cucumber plants infested by two-spotted spider mites. **a** Visible damage as chlorotic spots in cucumber accession Chinese long (CL) after 1, 2 or 3 days of TSSM infestation; **b** damaged area as percentage of the total leaf area (means ± SD; N = 3) in bitter accession Chinese long (CL, grey bars) and non-bitter accession Corona (CO, white bars); **c** mean (± SD) number of eggs per adult female mite per day produced on leaf discs of Chinese long (CL) or Corona (CO) (N = 30 leaf discs with two female mites); **d** volatiles emitted by cucumber leaves that were either non-infested or infested with spider mites for 1, 2 or 3 days. *CO* open circles, *CL* closed circles. GLVs represent the sum (*E*)-2-hexenal, (*Z*)-3-hexen-1-ol and (*Z*)-3-hexenyl acetate, monoTP represent the sum of monoterpenes α-pinene, limonene, (*E*)-β-ocimene, an unidentified monoterpene and linalool, sesquiTP represent the sum of sesquiterpenes (*E*)-β-caryophyllene, α-bergamotene, an unidentified sesquiterpene and (*E,E*)-α-farnesene and DMNT + TMTT is the sum of homoterpenes (*E*)-4,8-dimethyl-1,3,7-nonatriene and (*E,E*)-4,8,12-trimethyltrideca-1,3,7,11-tetraene. Data represent mean ± SD; N = 5; **e** relative attraction of predatory mites towards the odour blends of TSSM-infested CL and CO plants compared to non-infested plants and to each other. Data represent mean ± SD; N = 6. Differences between genotypes in percentage of damage, oviposition and volatiles on consecutive days were tested for significance using ANOVA after Levine’s test for equal variance. Predator preferences were tests using Chi-square test. *ns* not significant, *P < 0.05, **P < 0.01, ***P < 0.001

Damage as a result of TSSM feeding was visible as chlorotic spots (Fig. 1a) from 2 days after introduction of mites onwards and total chlorotic area reached 5.6% of the leaf in accession Chinese long 9930 (CL) and 8% in Corona (CO) after 3 days (Fig. 1b). Mites performed better on CO than on CL as more eggs per female were deposited over a time span of 7 days in a leaf disc assay (Fig. 1c).

TSSM infestation increased volatile emission in both accessions. Green Leaf Volatiles (GLVs)—including (*E*)-2-hexenal, (*Z*)-3-hexen-1-ol and (*Z*)-3-hexenyl acetate—rapidly increased on the first day post infestation followed by a small decrease in the next 2 days and was stronger in CO compared to CL (Fig. 1d). Methyl salicylate was emitted in small amounts by CL only and increased continuously during the 3 days post infestation (data not shown). Emission of monoterpenoids—including α-pinene, limonene, (*E*)-β-ocimene, an unidentified monoterpene and linalool—gradually increased from the onset of infestation, while sesquiterpene emission—including (*E*)-β-caryophyllene, α-bergamotene, (*E,E*)-α-farnesene and an unidentified sesquiterpene—increased from day 2 onwards. While the increase was comparable in both accessions, the absolute emission of mono- and sesquiterpenoids was higher in CO. Emission of homoterpenes DMNT and (*E,E*)-4,8,12-trimethyltrideca-1,3,7,11-tetraene (TMTT) increased strongest, and together they were emitted at even higher levels than the sum of mono- and sesquiterpenoids together (Fig. 1d). In both accessions homoterpenes were the dominant compounds in the induced volatile blend after 3 days of TSSM infestation. Predatory mites were attracted to the volatiles emitted by TSSM-infested plants. The relative attractiveness of both accessions increased with progressing infestation, while the blend of 3-day TSSM-infested CO attracted more predatory mites compared to that of 3-day TSSM-infested CL (Fig. 1e).

To determine the time frame of early defence responses and hence select time points for RNA-seq analysis, we analysed *LIPOXYGENASE* (Csa2M024440) and (*E,E*)*-α-FARNESENE SYNTHASE* (Csa3M095040) expression, two genes known to be induced by TSSM and involved in the biosynthesis of volatile compounds (Mercke et al. 2004). Comparison of non-infested and TSSM-infested CL leaves showed that transcripts of both genes were not significantly induced within the first 15 h after the onset of infestation (Supplemental Fig. S1). After 24 h, *LIPOXYGENASE* transcripts increased, whereas (*E,E)-α-FARNESENE SYNTHASE* expression increased at 3 days after infestation, corresponding with (*E,E*)-α-farnesene emission. As we have predominantly interest in adaptation of the metabolome, we analysed overall gene expression after 24, 48 and 72 h of TSSM infestation.

### Induced cucumber transcriptional responses to TSSM feeding

As we have interest in the early responses of cucumber leaves to TSSM infestation, plants were infested with a low density of mites, to more or less mimic a normal dispersal scenario. Since mites were not removed from the leaves when these were harvested, we aligned the clean reads from the RNA-seq analysis to the TSSM genome (Grbic et al. 2011) resulting in max 1% of the reads that could be mapped to the TSSM genome (data not shown), while more than 80% of the reads could be mapped to the cucumber genome (Supplementary Fig. S2). In summary, the assessment suggests that the obtained sequencing data are representative of the changes in the cucumber transcriptome during the time-course experiment (Supplemental Fig. S2). Validation of RNA-seq transcript data by analysing expression patterns of selected genes by qRT-PCR indicated comparable expression patterns during the time span of our experiment and we concluded that the gene expression data we obtained form a good basis for further analysis (Supplemental Fig. S3).

**Fig. 2:**
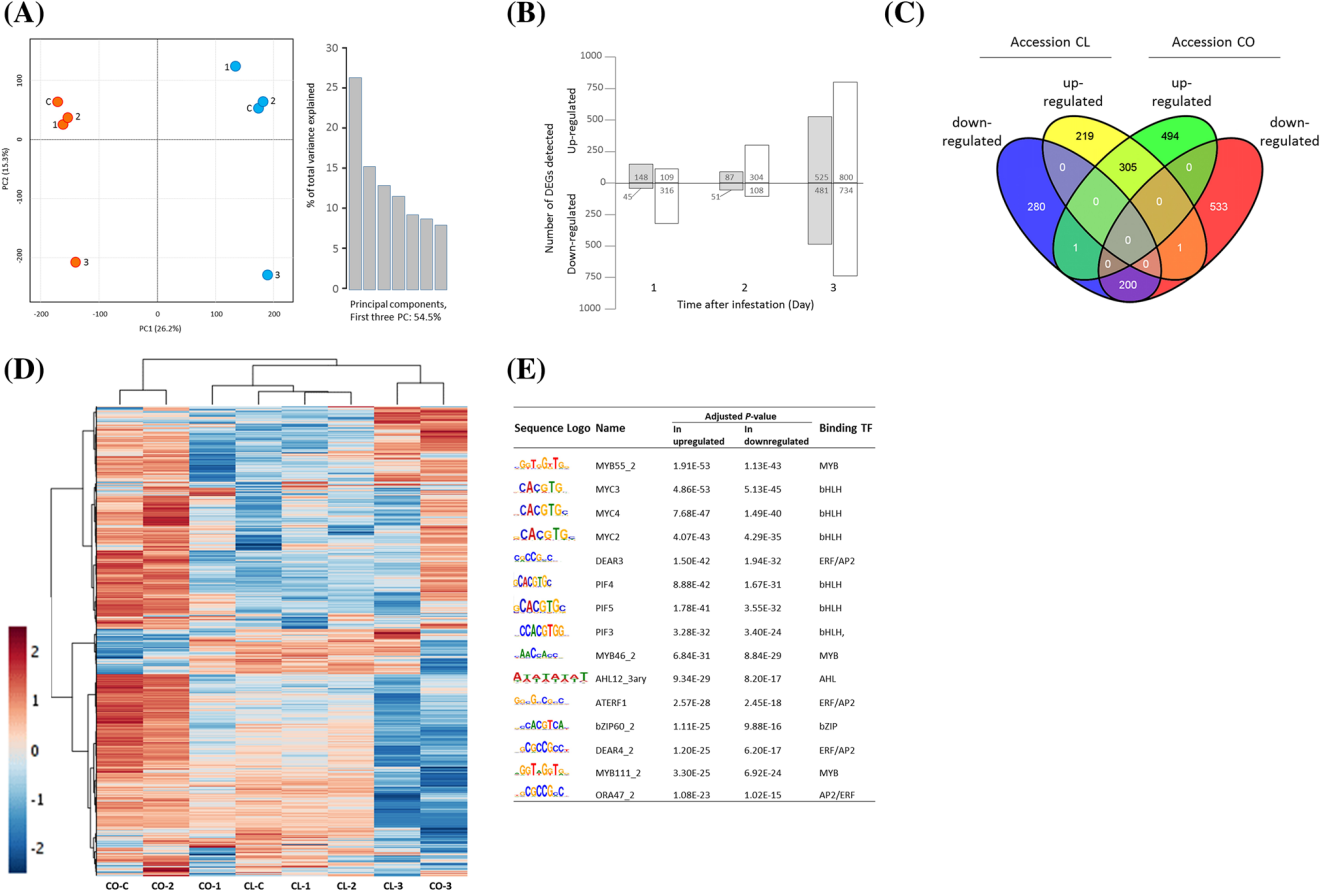
Transcriptional responses to TSSM infestation in two cucumber accessions. **a** Principal component analysis (PCA) on RPKM values of gene transcripts (normalized to sum, log2-transformed, and Pareto scaled) found present in leaves of cucumber Chinese long (CL, red) and Corona (CO, blue) that were infested with TSSM for 1, 2, or 3 days or were left uninfested (**c**); **b** differentially expressed genes [log2(infested/non-infested) ≥ 1, FDR < 0.01] detected upon TSSM infestation; **c** Venn diagram showing overlapping and different DEGs from accessions CL and CO in leaves that were infested with TSSM for 3 days; **d** heat map depiction of all differentially expressed genes between TSSM infested and non-infested plants. Blue represents low expression and red represents high expression compared to the average expression of each gene during the infestation; **e** the top15 putative TF binding motifs enriched in the 2kB promoter regions of upregulated and downregulated genes compared to random sequences

Unbiased principal component analysis (PCA) using Log_2_(RPKM) values showed that the first two principal components explain 46.2% of the total variation corresponding to the effect of both accession and TSSM (Fig. 2a). Subsequently, 2348 DEGs were identified by comparing gene expression at every time point of infestation with that of the non-infested sample for both accessions (Supplemental Table S2). Two days after the onset of infestation, less than 200 DEGs were identified in CL, whereas in CO more than 400 DEGs were found of which 304 were up-regulated and 108 were down-regulated compared with non-infested leaves (Fig. 2b). For both accessions the number of DEGs strongly increased with progressing infestation to 1006 in CL and 1534 in CO after 3 days. Accessions shared 505 DEGs 3 days after infestation, representing half of all DEGs in CL and almost a third in CO (Fig. 2c). Around 60% of the commonly identified DEGs were found to be up-regulated and 40% were down-regulated in both accessions. Hierarchical cluster analysis based on Pearson correlation of DEGs confirmed that within the first 2 days of infestation differences in gene expression between accessions are larger than changes in expression as caused by TSSM feeding (Fig. 2d, Supplemental Table S4). Three days after the onset of infestation, the herbivores became the major driving force for expression differences and overruled expression differences between accessions.

Analysis of the 2 kB upstream sequences of DEGs identified motifs that are enriched upon TSSM infestation. MYC2, MYC3, and MYC4 elements, all binding sites of MYC TFs, MYB55_2 element binding to MYB TFs and DEAR3 binding to ERF TFs were the five most enriched motifs found (Fig. 2e). Furthermore, PHYTOCHROME INTERACTING FACTORs PIF4, PIF5 and PIF3 motifs that can bind to bHLH TFs, MYB and ERF/AP2 binding motifs were found to be enriched as result of TSSM infestation.

**Fig. 3:**
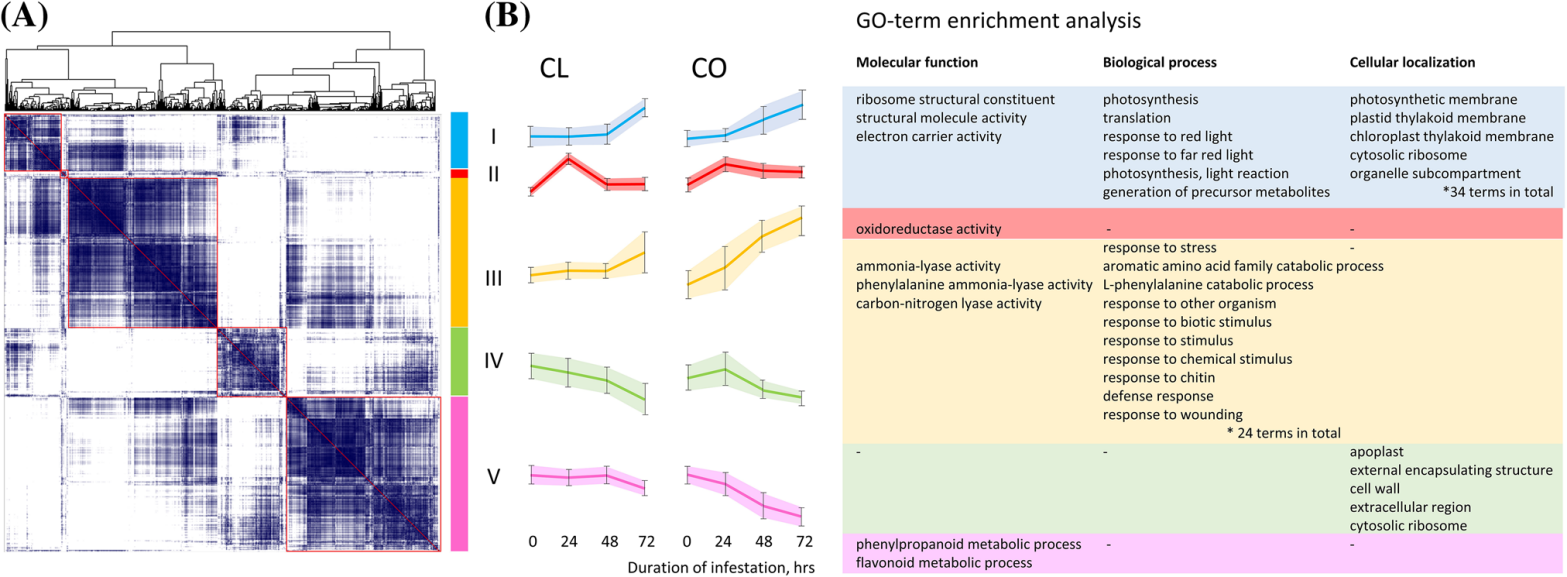
Similarity matrix of TSSM induced DEGs in Chinese long and Corona cucumber leaves. **a** Pearson correlation matrix of gene transcripts, the darker the colour, the higher the similarity of expression patterns. Red rectangles indicate clusters of genes that display strongly correlated expression patterns; **b** general expression trend of the genes in each cluster from A for each of the accessions and Gene Ontology terms enriched in each cluster. Enrichment analysis was performed using a hypergeometric test. *P* values were calculated using the Hochberg false discovery rate multi-test adjustment (≤ 0.05) as a threshold

A similarity matrix of gene expression patterns based on Pearson correlations showed that DEGs could be divided into five major clusters (Fig. 3). Cluster II contains only 29 genes that show quite a different expression pattern compared to all other clusters as expression of these genes was highly up-regulated after 1 day of infestation and decreased on day 2 and 3 compared to the non-infested control. Confirmation of the expression pattern in time was found for Csa1M589140, Csa2M055050 and Csa7M061710 using qRT-PCR (Supplemental Fig. S3). In cluster II only GO category ‘*oxidoreductase activity*’ was enriched (8 out of 29 genes). While our data are too limited to allow reliable conclusions, and we cannot distinguish between plant or mite-derived gene transcripts, interestingly, NADH:ubiquinone oxidoreductases are present in the saliva of TSSM (Bajda et al. 2017; Jonckheere et al. 2016). As oxidoreductases are known to catalyse redox reactions and known to suppress plant defences, other DEGs in cluster II DEGs might be associated with perception of attack. Cluster III includes 817 genes of which expression was induced from day 1 onwards and continued to increase over 3 days in both CO and CL (Fig. 3). Upregulated GO biological processes include those related to ‘*response to biotic stimulus*’, ‘*wounding and jasmonic acid*’ and also metabolic and catabolic processes including ‘*secondary metabolites*’, ‘*aromatic amino acids*’, ‘*L-phenylalanine’*, ‘*carbohydrates*’, indicating that DEGs in this cluster are related with transcriptional responses of signalling and first metabolite reconfiguration (Fig. 3, Supplementary Table S3). GO-term analysis did not yield any enrichment of cellular components and for molecular functions only ‘*phenylalanine ammonia-lyase activity*’ was found enriched. Cluster I contains 302 genes of which expression increased on day 3 and that were expressed higher in CL than in CO (Fig. 3b). GO enrichment showed that biological processes related with ‘*response to red and far-red light*’, ‘*photosynthesis*’ and ‘*translation*’ were enriched and furthermore processes related with ‘*generation of precursor metabolites and energy*’. Molecular functions of these genes are classified as ‘*electron carrier activity’* and ‘*structural constituent of ribosom*e’ whereas cellular component analysis showed that the GO-terms related to ‘*photosynthetic membrane*’, ‘*photosystem’*, ‘*thylakoid’*, ‘*plastid’*, ‘*nucleolus’* and ‘*ribosome’* were significantly enriched (Fig. 3, Supplementary Table S2). Clusters IV (371 genes) and V (829 genes) represent genes of which expression was suppressed by TSSM infestation compared with non-infested controls (Fig. 3). In group IV suppression is strongest in accession CL, while in group V it is strongest in CO. In group IV cellular components of ‘*cell wall*’, ‘*cytosolic ribosome*’ and ‘*apoplast*’ were enriched but no GO-terms for biological processes nor for molecular functions were significantly enriched. DEGs in group V were enriched for ‘*phenylpropanoid- and flavonoid metabolic processes*’, suggesting that TSSM might have a negative effect on these metabolic classes. It seems that the different clusters represent the temporal order of events, starting with perception of mite-attack (Cluster II), followed by signalling events and first alteration of the metabolome (Cluster III) and finally changes in the primary and specialized metabolome in both positive (Cluster I) and negative directions (Cluster IV and V). To obtain more insight in how different metabolic pathways that were found enriched in the GO-term analysis interact with each other, TSSM-induced DEGs were mapped to the KEGG pathway database (Supplementary Fig. S4). Three days after the onset of infestation several pathways were significantly influenced, including ‘*ribosome’*, ‘*protein export’*, *photosynthesis*, the ‘*phagosome pathway’* and *sesquiterpenoid* and *triterpenoid biosynthesis*. Only just not significant were *diterpenoid biosynthesis* and *terpenoid backbone biosynthesis*. Pathways that were significantly altered already on day 1 after infestation included *phenylpropanoid biosynthesis*, *phenylalanine metabolism* and *linoleic acid metabolism*.

**Fig. 4:**
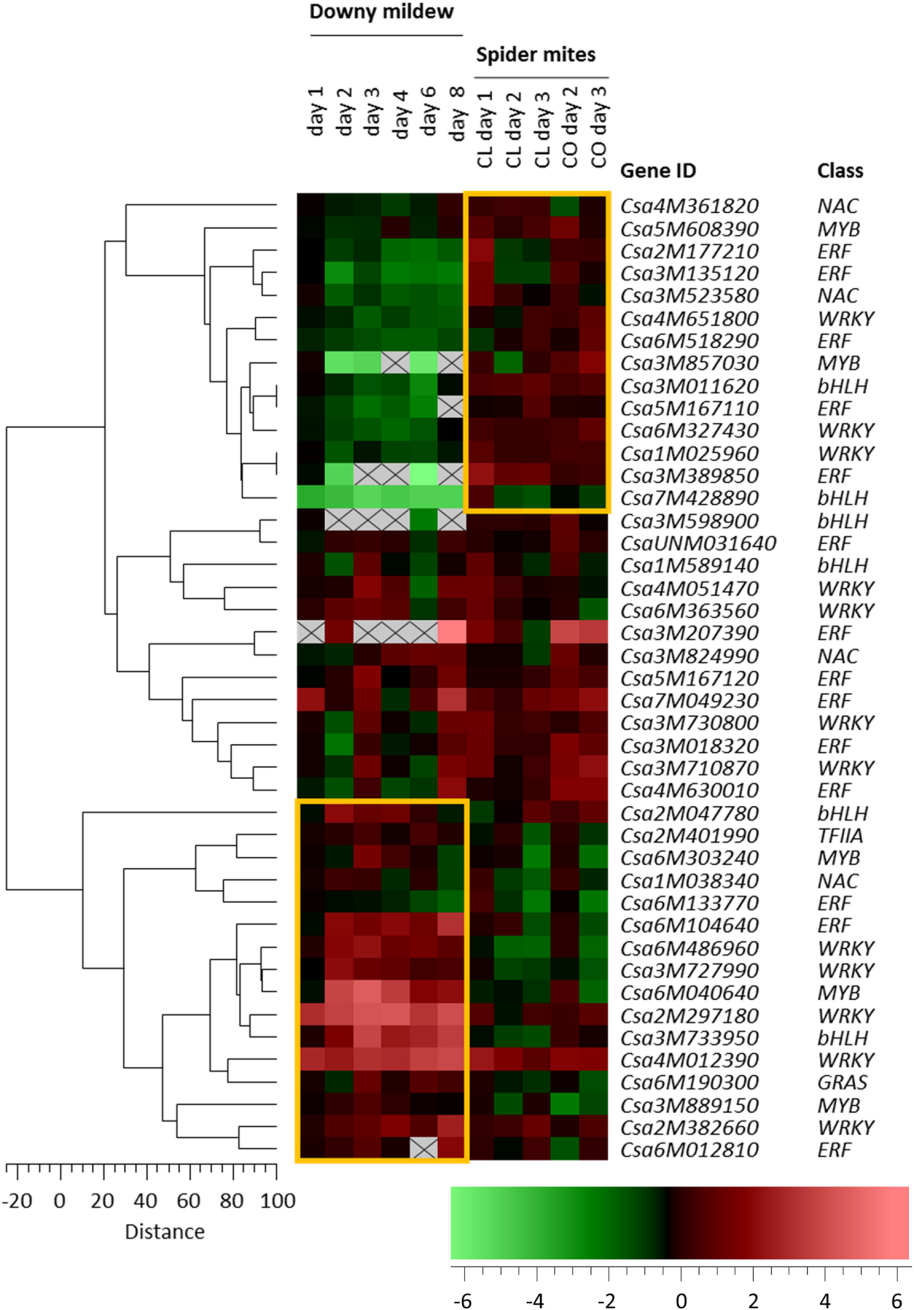
Hierarchical clustering analysis of transcription factors regulated by spider-mite feeding and downy-mildew infection according to the expression profile over different time points in each experiment. Expression was normalized as log 2 (day n/day 0), n = 1, 2 or 3 for the spider-mite experiment and 1, 2, 3, 4, 6 and 8 for the downy-mildew data set extracted from a published dataset (Adhikari et al. 2012). Colour coding: green represents low expression; red represents high expression. Grey areas with cross indicate that no transcripts were detected. Yellow rectangles indicate blocks of genes that have particularly high similarity in expression

To identify TFs that might play a role in early responses towards TSSM, we retrieved the 1212 genes from the cucumber genome with a putative TF function. Transcription of 119 TF genes was differentially regulated by TSSM infestation, of which 60 were generally upregulated and 59 were downregulated (Table 1). Among these 119 TF genes, the WRKY family was relatively most represented as 21% of the WRKY genes putatively present in the cucumber genome were differentially expressed upon TSSM infestation, followed by AP2/ERF (16%), bHLH (14%) and MYB (10%) TF genes. Considering the general trends of TF gene expression among DEGs, ERFs were preferentially upregulated and WRKYs preferentially downregulated upon TSSM herbivory. For the other TF families, fewer genes were found to be regulated by TSSM infestation as they were randomly distributed over induced and repressed DEGs. We compared our results with the transcriptional response of cucumber towards the pathogen downy mildew (DM, *Pseudoperonospora cubensis*) as described by Adhikari et al. (2012) to study similarities and differences in the involvement of TFs in the response of cucumber to two different biotic stresses. DM infection resulted in more TF gene families with DEGs than TSSM infestation, of which WRKY, NAC and GRAS were among the strongest affected TF genes upon DM infestation. Comparing both datasets resulted in 46 TF genes that were differentially expressed as a result of DM infection as well as TSSM infestation, although not always in the same direction (Fig. 4).

**Table 1:** Number of transcription factors belonging to different classes/families that are differentially regulated by two-spotted spider mites

	TSSM			
	Up	Down	Total no	%^a^
MYB	10	9	19	10
bHLH	11	10	21	14
AP2/ERF	17	7	24	16
C2H2	5	5	10	8
NAC	3	4	7	8
bZIP	1	2	3	4
WRKY	5	9	14	21
MADS	0	1	1	2
GRAS	0	3	3	8
DOF	0	3	3	8
HSF	0	1	1	3
TCP	1	0	1	4
GATA	1	0	1	4
NF-Y	0	2	2	8
ARF	1	0	1	10
HOX	1	1	2	12
SBP	0	0	0	0
Others	4	2	6	6
Total	60	59	119	10

The categories were in order with the size of each category in the genome

*Nr* number

^a^Percentage of each category that is affected by the indicated stress

### Genes involved in biosynthesis of terpenoids are regulated by TSSM

**Fig. 5:**
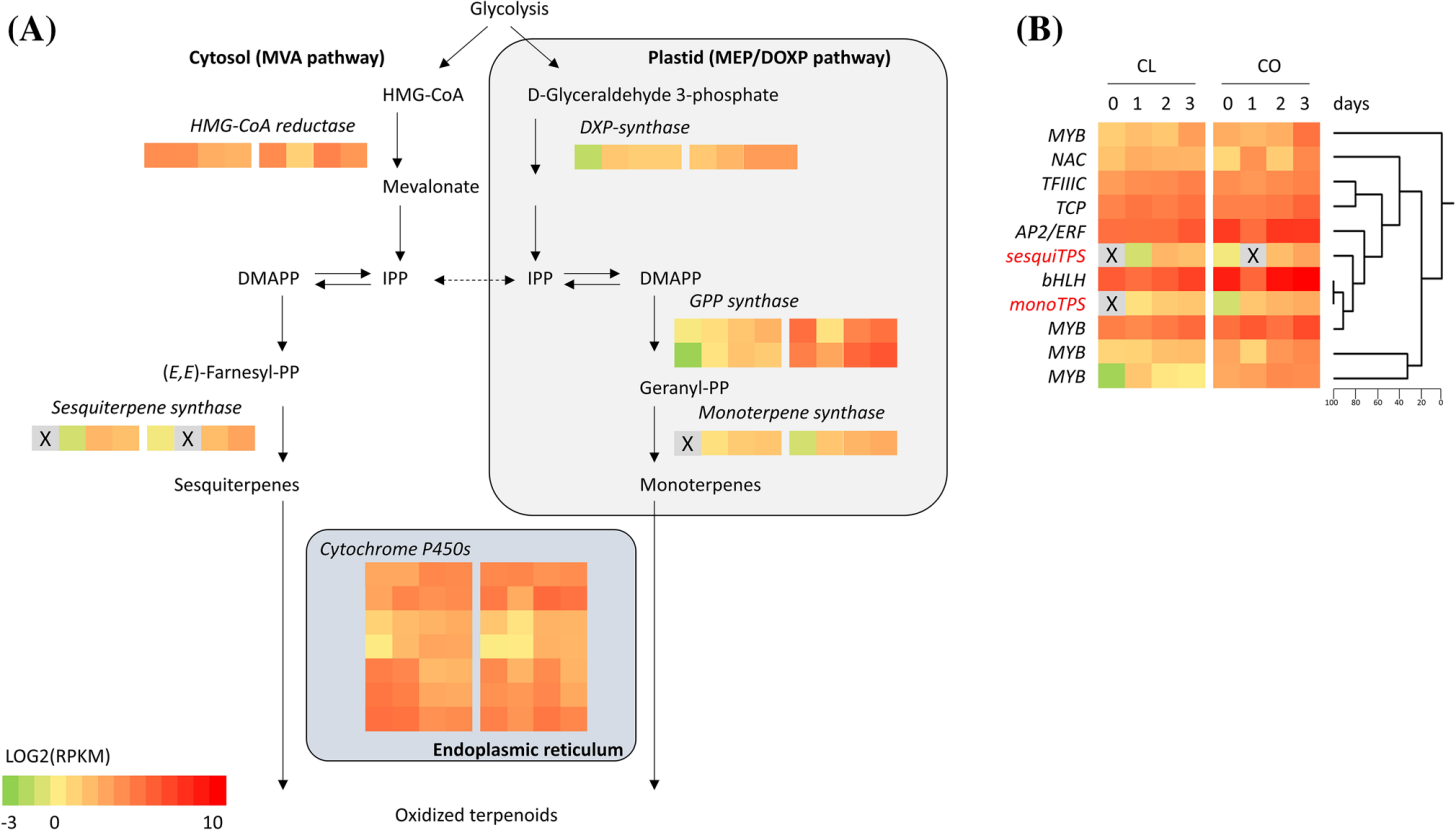
Terpenoid biosynthesis and regulation. **a** Relative expression of associated genes in accessions Chinese long (CL) and Corona (CO) infested by spider mites for different time periods. For each gene, the blocks represent expression levels in CL and CO day 0, 1, 2, 3 from left to right. **b** Co-expression of a mono- and a sesquiterpene synthase gene and TF genes during infestation. Grey areas with cross indicate that no transcripts were detected. DXP-synthase Csa1M420340; GPP synthase Csa6M487640, Csa7M211090; mTP synthase Csa2M299880; HMG-CoA reductase Csa7M029390; sTP synthase Csa3M095040; Cytochrome P450 Csa3M852560, Csa2M425750, Csa3M853160, Csa4M641760, Csa6M088160, Csa6M088170, Csa3M698490; MYB Csa3M183960, Csa7M000020, Csa4M638510, Csa6M121970; NAC Csa1M165230; TFIIIC Csa2M406680; TCP Csa3M564320; AP2/ERF Csa5M167110; bHLH Csa2M047780

GO and KEGG analysis revealed terpenoid-related processes and pathways dominantly present among DEGs induced by TSSM, including ‘*terpene metabolic processes’*, ‘*terpenoid backbone biosynthesis*’, ‘*sesquiterpenoid and triterpenoid biosynthesis’*, and ‘*limonene and pinene degradation*’ (Supplementary Fig. S4). Five DEGs were assigned the GO term of ‘*terpenoid backbone biosynthesis*’. Csa1M420340 encoding DXP-synthase (1-DEOXY-D-XYLULOSE-5-PHOSPHATE SYNTHASE) catalysing the rate limiting enzymatic step in the plastidic MEP/DOXP pathway that supplies the substrate for mono- and diterpene biosynthesis, was upregulated (Fig. 5a). In addition, the strongest TSSM induced TF, i.e. MYC (Csa3M002860) co-expressed with DXP.

Csa6M487640 and Csa7M211090 both encoding GERANYL DIPHOSPHATE SYNTHASE were upregulated in CL, while in CO this was true only for Csa7M211090. In contrast, despite the enrichment of ‘*sesquiterpene metabolic process’*, gene Csa7M029390 encoding 3-HYDROXY-METHYLGLUTARYL COENZYME A REDUCTASE catalysing the rate limiting step in the cytosolic mevalonate pathway to produce the substrates for sesquiterpene and triterpene biosynthesis was down-regulated in both accessions during early mite infestation.

Expression of Csa2M299880—encoding a putative monoterpene synthase—and Csa3M095040—encoding (*E,E*)-α-farnesene synthase (Mercke et al. 2004)—was not or hardly detected in non-infested plants and was up-regulated during the first 3 days of infestation in both accessions. In addition, expression of the putative monoterpene synthase Csa1M066560 was induced in CL and those of Csa1M068570 and Csa2M298300 in CO. A small increase in expression of (*E*)-β-caryophyllene synthase (Csa3M097040, Mercke et al. 2004) was detected in TSSM-infested CO leaves, but not in CL. The upregulation of the expression of these genes coincided with mono- and sesquiterpenoid emission, as (*E*)-β-ocimene, α-pinene, and linalool were found to be present in the blend from TSSM-infested plants from day 1 onwards, while (*E,E*)-α-farnesene and (*E*)-β-caryophyllene were emitted from day 2 after infestation (Fig. 1). Strongest TSSM-induced terpene synthase genes (Csa2M29988 and Csa3M09504) closely co-expressed with each other and with a bHLH (Csa2M047780), a MYB (Csa7M000020) and an ERF TF (Csa5M167110) (Fig. 5b).

Three of the seven P450 genes, which were mapped onto the pathway of ‘*limonene and pinene degradation*’ and hence might be involved in conversion of terpenes into more oxidised terpenoids, were up-regulated in both accessions after infestation. Two of these (Csa3M853160; Csa3M852560) have high homology to CYP82G, which in Arabidopsis is known to be involved in the conversion of nerolidol to DMNT and geranyl linalool to TMTT (Lee et al. 2010). Further studies must resolve whether these cucumber P450s are indeed responsible for the formation of TSSM-induced homoterpenoids in cucumber. In contrast, three other P450s in this group were suppressed by TSSM infestation, with their annotations suggesting a role in flavonoid (Csa6M088160) and triterpene (Csa3M698490) biosynthesis.

### TSSM suppress expression of genes associated with biosynthesis of cucurbitacin C

**Fig. 6:**
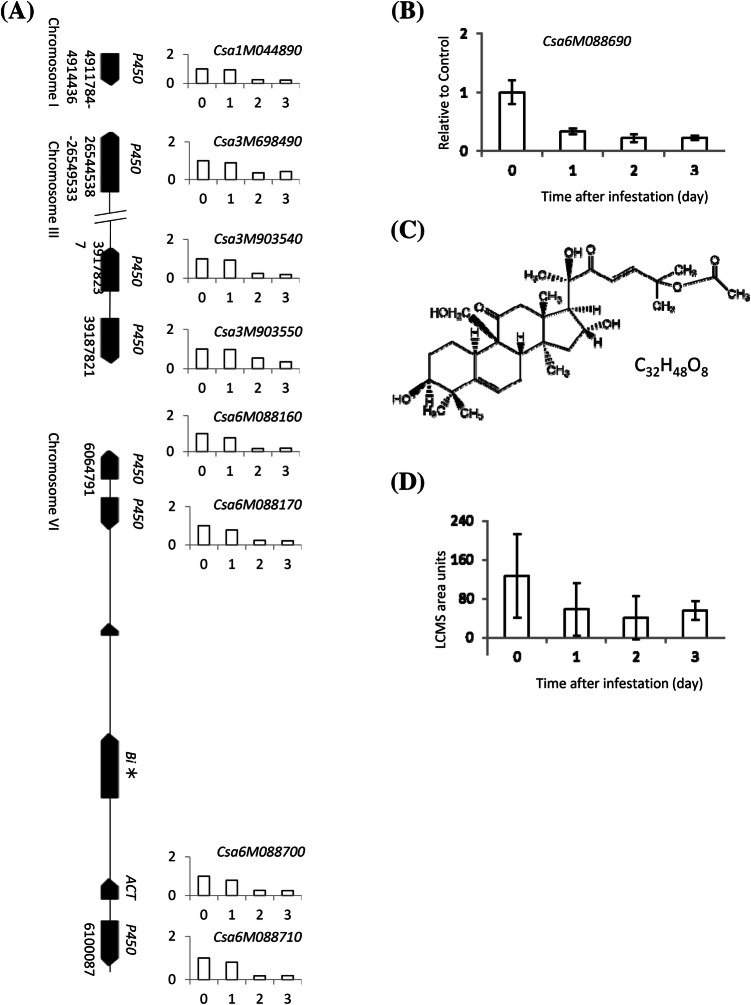
Relative expression of genes associated with biosynthesis of cucurbitacin C in spider-mite infested cucumber leaves. **a** Transcripts of genes involved in cucurbitacin C biosynthesis (Shang et al. 2014) in leaves of accession Chinese long after infestation with spider mites for 1, 2 or 3 days, for reasons of clarity only the mean RPKM value is shown; **b** qRT-PCR analysis of Bi (Bitter) gene (Csa6M088690) transcripts in spider-mite infested leaves of accession Chinese long (mean ± SE, N = 3), no transcripts were detected in accession Corona; **c** molecular structure of cucurbitacin C; **d** concentration of cucurbitacin C in cucumber Chinese long leaves that were infested by spider mites for 1, 2 or 3 days or were left untreat

Nine genes functional in the cucurbitacin C biosynthetic pathway were previously identified (Shang et al. 2014) and transcripts of all except one were detected in our RNA-seq data. Interestingly, the expression of these eight genes was down regulated after TSSM infestation in both the bitter accession CL and the non-bitter accession CO (Fig. 6a). Expression of OXIDOSQUALENE CYCLASE (Csa6M088690, Bi) known to confer bitterness, was not detected in our RNA-seq experiment. However, qRT-PCR analysis for this gene revealed that transcripts are indeed present in CL and decreased after infestation (Fig. 6b). Furthermore, the amount of cucurbitacin C in TSSM-infested leaves of accession CL showed a negative (though not significant) trend with progressing infestation (Fig. 6d), while no cucurbitacin C was detected in CO. Seven cucurbitacin associated genes were found co-expressed with a gene encoding a bHLH TF (Csa1M051740), which has not been described previously, suggesting a role in the early steps of cucurbitacin biosynthesis. This TF was assigned GO term ‘*response to gibberellin stimulus*’.

## Discussion

Recognition of the attacker leads to a transient reconfiguration of the plants’ transcriptome in such a way that, amongst others, its metabolome is altered in response to the specific attacker. Identification of responsive genes and determining their regulation is a central goal to improve our understanding of the responses of plants to biotic stresses. Here, we provide an analysis of genes whose transcription is affected by early TSSM herbivory on cucumber foliage. In the first 3 days of infestation, genes involved in JA signalling, photosynthesis, biosynthesis of terpenoids and encoding LIPOXYGENASEs were upregulated. Genes associated with flavonoids and, surprisingly, genes associated with the biosynthesis of cucurbitacin C, which is an important anti-herbivore secondary metabolite in cucumber (Balkema-Boomstra et al. 2003), were suppressed during the first 3 days of spider mite infestation.

Early TSSM infestation at a relatively low infestation pressure resulted in about 10% of the genes being differentially expressed. Likewise, little more than 10% of the putatively annotated TF genes significantly changed expression upon TSSM infestation. As TFs are key regulators of plant gene expression and often connect (hormonal) signalling pathways to biosynthetic pathways, identification of TF genes among the TSSM-induced DEGs may help to understand the complexity of the defence regulatory networks. The divergence of the expression patterns of DEGs responsive to different organisms are expected to have consequences for the expression of the target genes of the TFs. However, the number of TF genes induced by both TSSM and DM was higher than the number that showed opposite expression patterns. These commonly upregulated TF genes are most likely involved in a shared basal response of cucumber to biotic stress. Similar, considerable overlap in transcriptional changes was induced in Arabidopsis by infection by the pathogenic leaf-infecting bacterium, *Pseudomonas syringae* pv. tomato, and the pathogenic leaf-infecting fungus *Alternaria brassicicola*, and feeding by the chewing caterpillar *Pieris rapae*, the cell-content feeding thrips *Frankliniella occidentalis* and the phloem-sucking aphid *Myzus persicae* (De Vos et al. 2005).

Enrichment analysis in the promoters of the DEGs implies that TSSM-induced DEGs are very likely recognized and regulated by MYB, bHLH and AP2/ERF TFs. WRKY, AP2/ERF, bHLH and MYB TF genes were most responsive to TSSM and are reported to encode important TFs in plant defence (Seo and Choi 2015). MYBs, bHLHs and ERFs were the most regulated TF gene families in TSSM-infested tomato representing about 8.8% of DEGs (Martel et al. 2015). About 21% of the genes encoding WKRYs in the cucumber genome were responsive to infestation, making the WRKYs the most affected TF family. WRKY TFs play a pivotal role in various developmental process, abiotic stresses and biotic stresses (Eulgem and Somssich 2007) of which their role in defence against plant pathogens is well documented while little is known about their role in defence against herbivory. WRKY3 and WRKY6 co-ordinately regulate defence in *N. attenuata* to herbivores in a JA-dependent way (Skibbe et al. 2008). In contrast to the relative contribution of TSSM-regulated WRKY TF genes in cucumber (14 out of 119 TF genes, 11.8%), only nine WRKYs (out of 187, 4.8%) were regulated in tomato in response to TSSM.

Induction of the expression of MYB TF genes in response to herbivory was observed in Arabidopsis (Gigolashvili et al. 2007) and *N. attenuata* (Kaur et al. 2010). Multiple identified MYB TFs affect biosynthesis of defensive secondary metabolites by regulating the expression of associated biosynthesis genes (Gigolashvili et al. 2007; Gális et al. 2006; Kaur et al. 2010; Onkokesung et al. 2014). Cucumber TF genes that co-expressed with CsTPSs include four MYBs. Furthermore, two MYBs have an almost similar expression pattern as CsLOX, suggesting that these MYBs play an essential role in the regulation of herbivore-induced volatile (terpenoids and GLVs) formation in cucumber.

The bHLH family in cucumber is smaller than the MYB family but relatively more genes in this family were responsive to TSSM. Functional characterisation of AtMYC2 (with a bHLH domain) showed that it can up-regulate a group of genes and suppress another group of genes that both are involved in the JA signalling pathway (Lorenzo et al. 2004; Dombrecht et al. 2007). Moreover, a triple mutant of Arabidopsis lacking functional AtMYC2, AtMYC3, and AtMYC4 became extremely susceptible to the generalist herbivore *Spodoptera littoralis* (Schweizer et al. 2013). MYCs can interact with each other as well as with other proteins such as JAZ to control the regulatory nodes of plant processes involved in induced defences (Fernandez-Calvo et al. 2011). Considering that the cucumber response to TSSM also involves changes in the concentration of JA and changes in the expression of JA-regulated genes, it suggests that the bHLHs identified among the DEGs may be involved in these processes.

Activation of JA signalling by TSSM has been widely observed in plants species including Arabidopsis (Zhurov et al. 2014), lima bean (Ozawa et al. 2000; Dicke et al. 1999), grape (Diaz-Riquelme et al. 2016) and tomato (Ament et al. 2004). Transcriptional changes in TSSM-infested Arabidopsis leaves suggest JA as the major hormone mediating inducible defence responses (Zhurov et al. 2014). In tomato, genes involved in the jasmonate pathway responded to TSSM infestation within 1 day and emission of volatile terpenoids and attractiveness to predators significantly increased after 4 days (Ament et al. 2004). Comparison of DEGs in the tomato *def-1* mutant, which is mutated in the ability to accumulate JA in response to wounding and herbivory (Howe et al. 1996), showed that approximately 95% of DEGs 24 h after infestation by TSSM are dependent on JA (Martel et al. 2015). Upon TSSM-infestation for 7 days, cucumber leaves emitted various volatiles not emitted by non-infested plants and JA treatment resulted in a virtually similar volatile blend that includes GLVs and terpenoids (Kappers et al. 2010). In addition, MeSA is emitted in small amounts in some cucumber genotypes after TSSM infestation while it is absent in the blend of others (Kappers et al. 2010). In Arabidopsis, BSMT1 encodes a SA CARBOXY-METHYLTRANSFERASE and expression in leaves is upregulated by JA and herbivory (Chen et al. 2003). In cucumber, three BSMT1 homologs were found but none of them was induced in either CL or CO, although MeSA was detected among the volatiles emitted from TSSM-infested CL leaves. The SA-related GO-term, ‘*salicylic acid metabolic process’*, was found enriched and includes DEGs encoding PAL catalysing the first step in the biosynthesis of metabolites with a phenylpropanoid skeleton from L-phenylalanine, including SA. PAL transcript accumulation increased after pathogen infection, wounding and UV light (Dixon and Paiva 1995). However, PAL is also involved in many other pathways and does not specifically indicate activity of the SA pathway. Furthermore, expression of SA marker genes EDS1 and PAD4 did not increase in CL during early infestation and was downregulated in CO. In contrast, PR1, a well-known SA-responsive marker gene was up-regulated in both accessions by TSSM. Increase of endogenous SA and the expression of downstream pathway genes was observed in lima bean upon TSSM infestation (Ozawa et al. 2000). SA also accumulated in Arabidopsis leaves infested with a very high TSSM density (Zhurov et al. 2014). Taken together, we only found weak indications that SA signalling is induced in cucumber in a normal dispersal scenario during the first days of infestation.

The leaf metabolome was altered as result of TSSM infestation as mono- and sesquiterpene emission increased while the amount of non-volatile triterpenoid cucurbitacin C decreased. TSSM-induced changes in expression of genes involved in biosynthesis of terpenoids biosynthesis and their precursors suggest that this chemical class of compounds plays an important role in the response of cucumber to TSSM infestation. Furthermore, transcripts of genes encoding cytochrome P450 enzymes putatively involved in terpenoid modification were also differentially regulated during TSSM-infestation. TSSM-induced TPSs and altered volatile profiles were reported for several plant species including cucumber (Kappers et al. 2010; Mercke et al. 2004). Quantitative and qualitative variation in emitted terpenoids was found in different cucumber accessions and coincides with different attractiveness to predatory mites (Kappers et al. 2010; 2011). Changes in the expression of CsTPSs together with the genes functional in synthesis of terpene precursors and terpene modification results in changes in the profile of terpenoids released by the plant and had consequences for the attractiveness towards predators.

Unexpectedly, genes associated with the biosynthesis of cucurbitacin C (Shang et al. 2014) were down-regulated during early TSSM infestation, coinciding with a decreasing cucurbitacin C content during the first days of infestation. Triterpenoid cucurbitacins confer a bitter taste to cucurbits including cucumber (cucurbitacin C) and are known to discourage multiple herbivores such as beetles, larvae of lepidopteran species, cockroaches and TSSM (Agrawal et al. 2002; Balkema-Boomstra et al. 2003, Tallamy et al. 1997). Spider-mite performance was indeed better on accession CO, which is a non-bitter genotype. Introduction of TSSM on the cotyledons of bitter cucumber resulted in an increase in the cucurbitacin C concentration in infested cotelydons and in the first systemic leaf 7 to 10 days after the mites were removed from the plants (Agrawal et al. 1999). Although by unknown reason no transcripts were detected in our RNA-seq for the Bi gene [of which the bi allele /mutation is responsible for the loss of bitterness (Shang et al. 2014)], transcript levels of the other eight genes in the cucurbitacin C pathway were reduced after introduction of TSSM in both accession CL with bitter leaves (Bi allele) and accession CO with non-bitter leaves (bi allele). Furthermore, qPCR for the Bi gene itself in CL showed a decrease in expression too, while no transcripts were detected in CO. At first sight these results appear to conflict with those reported by Agrawal et al. (2002) although the different time frames in both studies could be of importance. In the experiments by Agrawal and co-workers, mites were introduced on to cotyledons for 3 days and then removed using a miticide. Seven to ten days after that, the leaves (local and systemic) were collected and the concentration of cucurbitacin C measured. In our study, TSSM were placed on true leaves and samples were collected on 1, 2 and 3 days after infestation. It is possible that the expression of genes involved in cucurbitacin C biosynthesis and cucurbitacin C content only decreased locally, which we analysed, while they increase systemically, in organs more important to protect (e.g. younger leaves). Furthermore, different *T. urticae* lines could differently trigger plant JA defence as demonstrated in tomato (Kant et al. 2008). What is more, *Tetranychus evansi* can manipulate tomato plant defence by reducing the formation of induced defence compounds such as proteinase inhibitors while herbivory of *T. urticae* did induce these defence responses (Sarmento et al. 2011). Whether *T. urticae* indeed specifically and locally represses direct defence in cucumber and/or whether this is specific for the mite strain used in our work, needs more in-depth study.

A bHLH TF was found to be co-expressed with the majority of cucurbitacin C biosynthesis associated genes in cucumber. Bi is regulated by two tandem bHLH genes, Bl (Bitter leaf, Csa5G156220), regulating Bi in leaves, and Bt (Bitter fruit, Csa5G157230), regulating Bi in fruits, respectively (Shang et al. 2014). Both these genes had low expression levels during the period of TSSM infestation and their expression trends did not correlate with the changes in cucurbitacin C content, indicating that these TFs are not responsive to biotic stresses. The presence of seven E-box motifs in the promoter region of Bi suggests bHLH TFs can affect Bi expression (Toledo-Ortiz et al. 2003) and hence the new identified bHLH TF is potentially regulating the expression of cucurbitacin biosynthesis genes upon biotic stress. In our current work, we aim to characterize the function of this new identified bHLH TF with respect to its role in adaptation of the cucumber leaf metabolome upon biotic stress.

In conclusion, we analysed the transcriptional response in *C. sativus* to the two-spotted spider mite *T. urticae*. Our study demonstrated that genes involved in JA signalling are significantly affected by spider-mite herbivory. Furthermore, genes associated with biosynthesis of volatile terpenoid and lipoxygenase-derived compounds which are involved in indirect defence, are upregulated while genes associated with biosynthesis of triterpene compound cucurbitacin C which is involved in direct defence are suppressed. These transcriptional changes match with the increased emission of terpenoids and green leaf volatiles and decreased content of cucurbitacin C in cucumber leaves. Furthermore, we have shown that MYB, bHLH, AP2/ERF and WRKY TFs were among the most regulated TFs during the first days of infestation by spider mites. Further work to confirm the involvement of these TFs in the induced indirect defence of cucumber is in progress [6862 words].

## Electronic supplementary material

Below is the link to the electronic supplementary material.

Supplementary Table S1: Primer 930 sequences used for qRT-PCR
Supplementary Table S2: DEGS detected in response to spider-mite feeding in cucumber
Supplementary Table S3: Cluster analysis and GO function of DEGs
Supplementary Table S4: Analysis of variance of DEGs (XLSX 345 kb)

Supplementary Table S5: Annotations of Transcription Factors (XLSX 117 kb)

Supplementary Figure S1: Transcript expression of CsLOX and CsFAR during early TSSM infestation
Supplementary Figure S2: Sequencing assessment of reads achieved from RNA-seq
Supplementary Figure S3: Validation of RNA-seq experimental data by qRT-PCR
Supplementary Fig. S4: Enriched KEGG pathways for spider-mite infested Chinese long cucumberleaves
Supplementary Fig. S5: Transcription factor co-expressed with Bi (Bitter) and related genes (PPTX 305 kb)

## Data Availability

The transcriptome data set supporting the results of this article is available through 10.17026/dans-xz7-ut4m. In addition, data sets supporting the results of this article are included as additional files.
