# Supplementary material for: Transcriptional and metabolite analysis reveal a shift in direct and indirect defences in response to spider-mite infestation in cucumber (Cucumis sativus)
Source: Plant Mol Biol. 2020 Apr 18;103(4):489–505. doi: 10.1007/s11103-020-01005-y (PMC7299927; doi:10.1007/s11103-020-01005-y)
Supplement: Supplementary file 3 — Supplementary Figure S1: Transcript expression of CsLOX and CsFAR during early TSSM infestation Supplementary Figure S2: Sequencing assessment of reads achieved from RNA-seq Supplementary Figure S3: Validation of RNA-seq experimental data by qRT-PCR Supplementary Fig. S4: Enriched KEGG pathways for spider-mite infested Chinese long cucumberleaves Supplementary Fig. S5: Transcription factor co-expressed with Bi (Bitter) and related genes (PPTX 305 kb) [file 11103_2020_1005_MOESM3_ESM.pptx]

## Slide 1
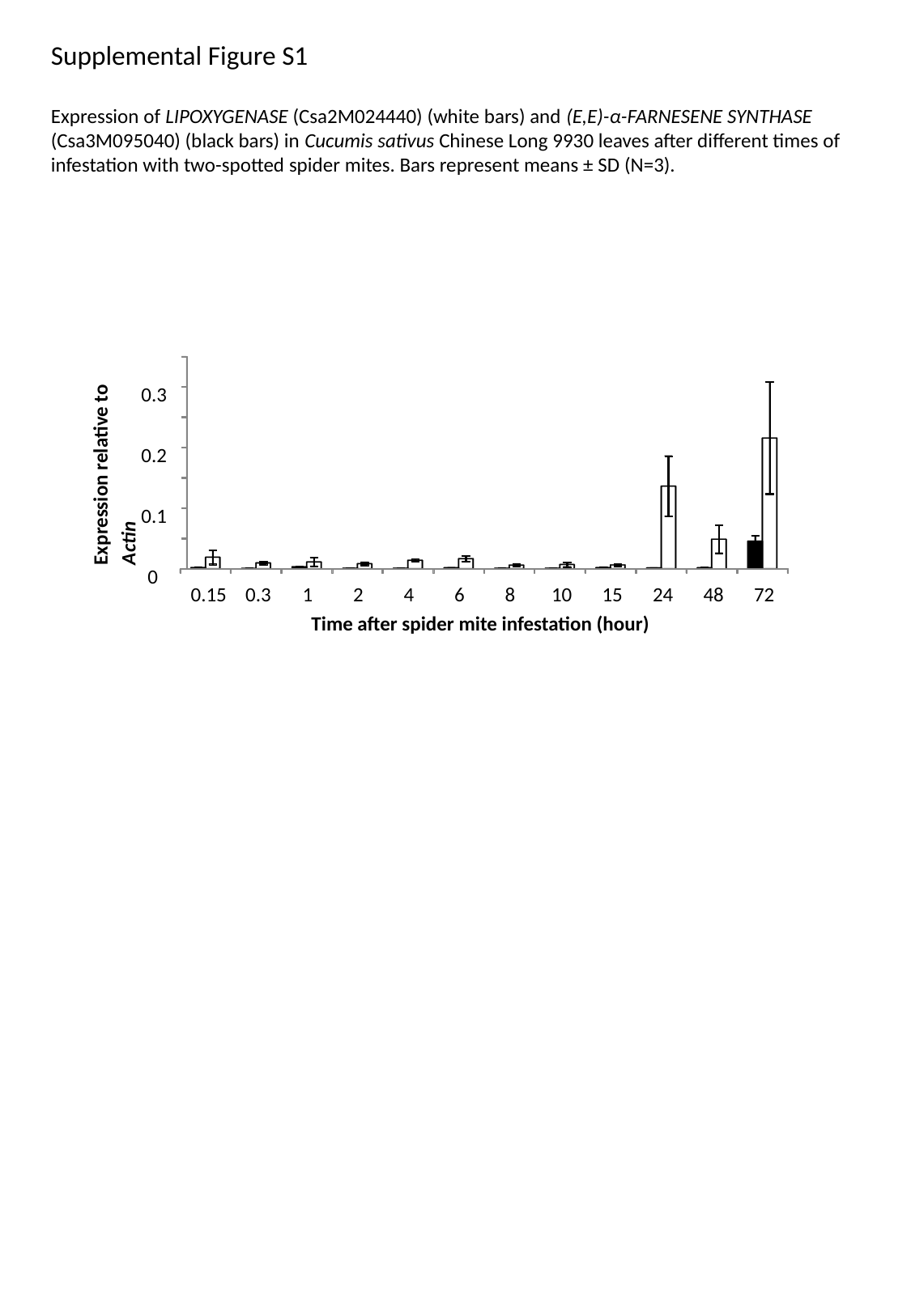

Supplemental Figure S1
Expression of LIPOXYGENASE (Csa2M024440) (white bars) and (E,E)-α-FARNESENE SYNTHASE (Csa3M095040) (black bars) in Cucumis sativus Chinese Long 9930 leaves after different times of infestation with two-spotted spider mites. Bars represent means ± SD (N=3).
Expression relative to Actin
0.3
0.2
0.1
0
0.15
0.3
1
2
4
6
8
10
15
24
48
72
Time after spider mite infestation (hour)

## Slide 2
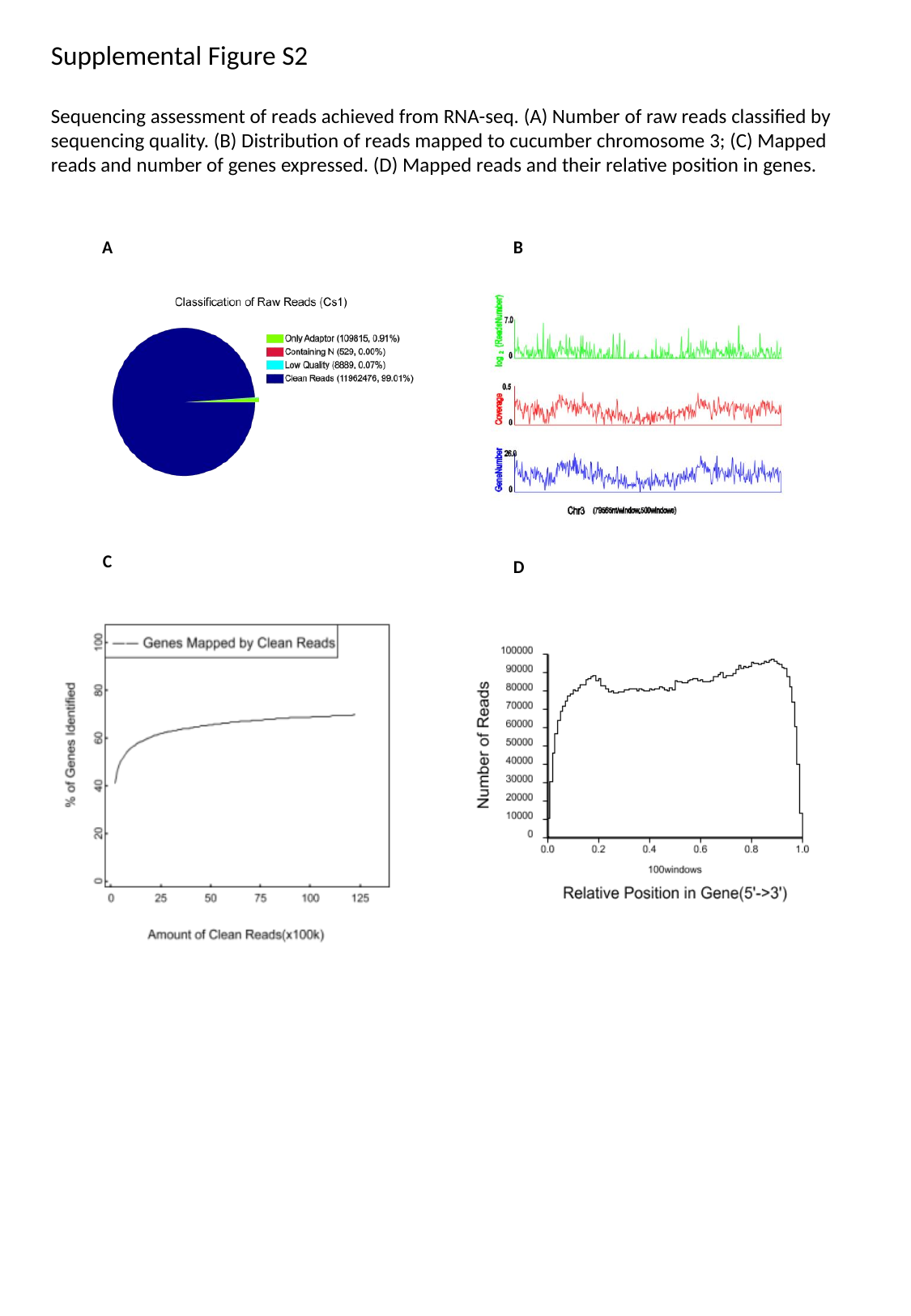

Supplemental Figure S2
Sequencing assessment of reads achieved from RNA-seq. (A) Number of raw reads classified by sequencing quality. (B) Distribution of reads mapped to cucumber chromosome 3; (C) Mapped reads and number of genes expressed. (D) Mapped reads and their relative position in genes.

## Slide 3
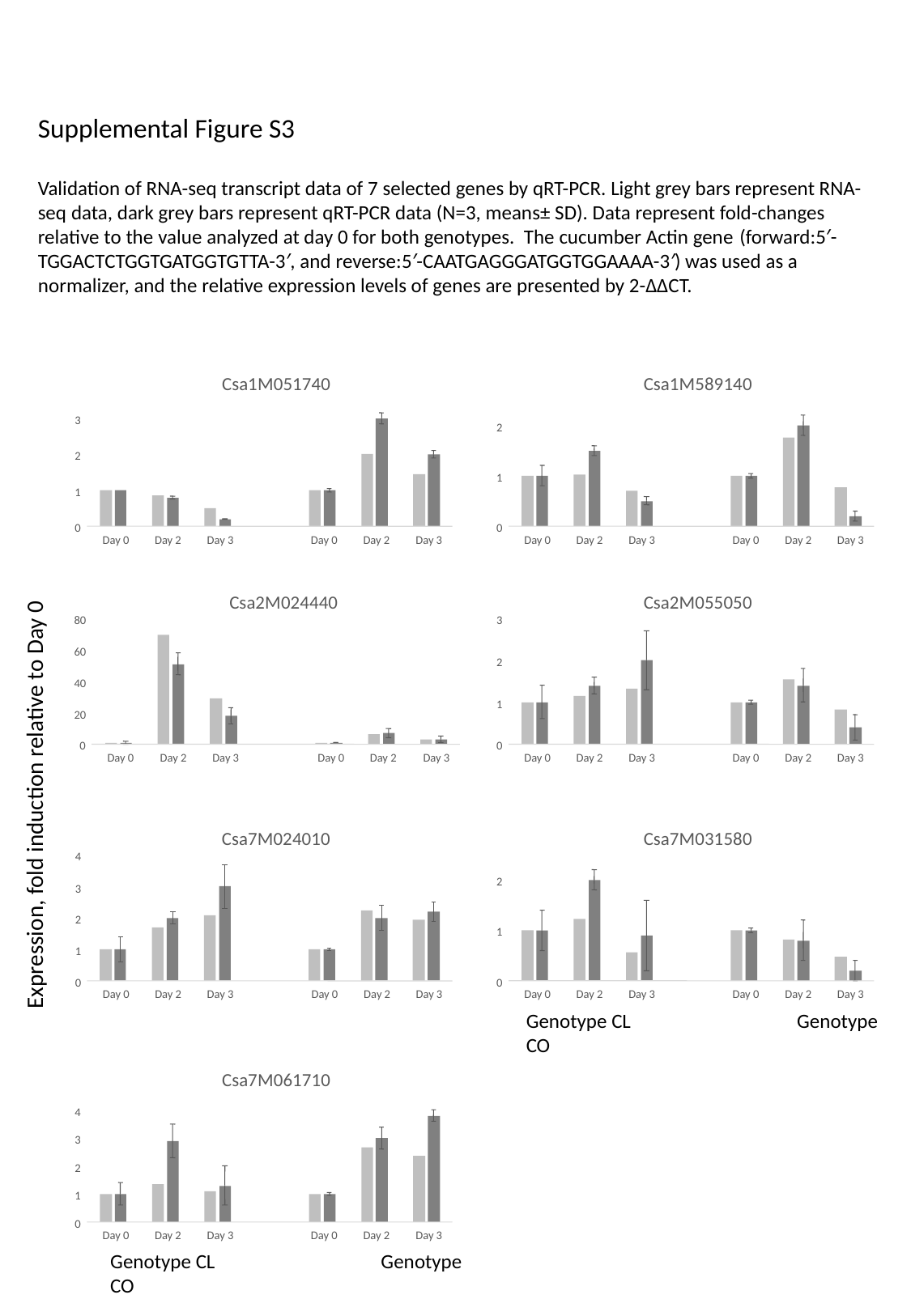

Supplemental Figure S3
Validation of RNA-seq transcript data of 7 selected genes by qRT-PCR. Light grey bars represent RNA-seq data, dark grey bars represent qRT-PCR data (N=3, means± SD). Data represent fold-changes relative to the value analyzed at day 0 for both genotypes. The cucumber Actin gene (forward:5′-TGGACTCTGGTGATGGTGTTA-3′, and reverse:5′-CAATGAGGGATGGTGGAAAA-3′) was used as a normalizer, and the relative expression levels of genes are presented by 2-ΔΔCT.
Csa1M589140
2
1
0
Day 0
Day 2
Day 3
Day 0
Day 2
Day 3
Csa1M051740
3
2
1
0
Day 0
Day 2
Day 3
Day 0
Day 2
Day 3
Csa2M024440
80
60
40
20
0
Day 0
Day 2
Day 3
Day 0
Day 2
Day 3
Csa2M055050
3
2
1
0
Day 0
Day 2
Day 3
Day 0
Day 2
Day 3
Expression, fold induction relative to Day 0
Csa7M024010
4
3
2
1
0
Day 0
Day 2
Day 3
Day 0
Day 2
Day 3
Csa7M031580
2
1
0
Day 0
Day 2
Day 3
Day 0
Day 2
Day 3
Genotype CL		 Genotype CO
Csa7M061710
4
3
2
1
0
Day 0
Day 2
Day 3
Day 0
Day 2
Day 3
Genotype CL		 Genotype CO

## Slide 4
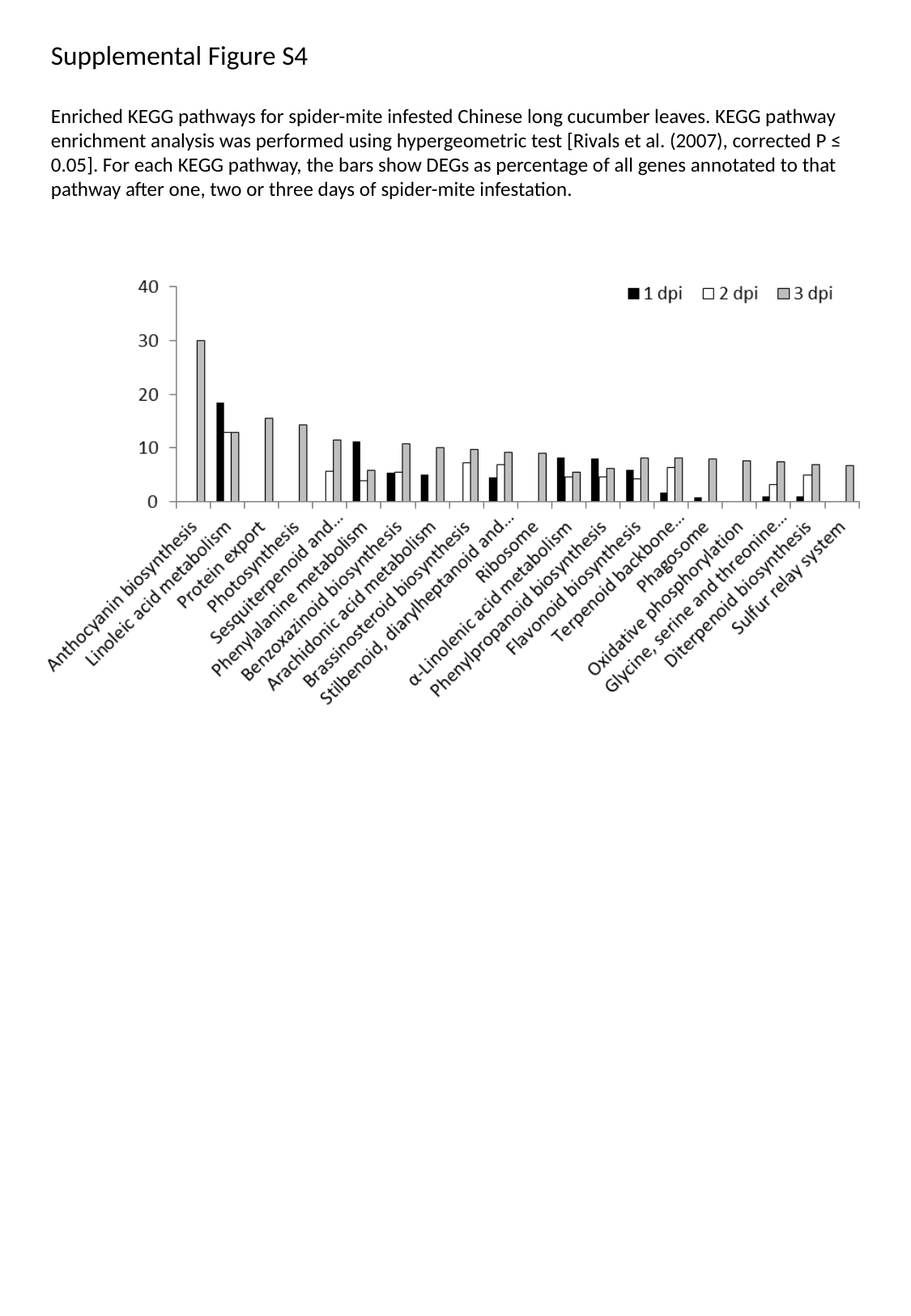

Supplemental Figure S4
Enriched KEGG pathways for spider-mite infested Chinese long cucumber leaves. KEGG pathway enrichment analysis was performed using hypergeometric test [Rivals et al. (2007), corrected P ≤ 0.05]. For each KEGG pathway, the bars show DEGs as percentage of all genes annotated to that pathway after one, two or three days of spider-mite infestation.

## Slide 5
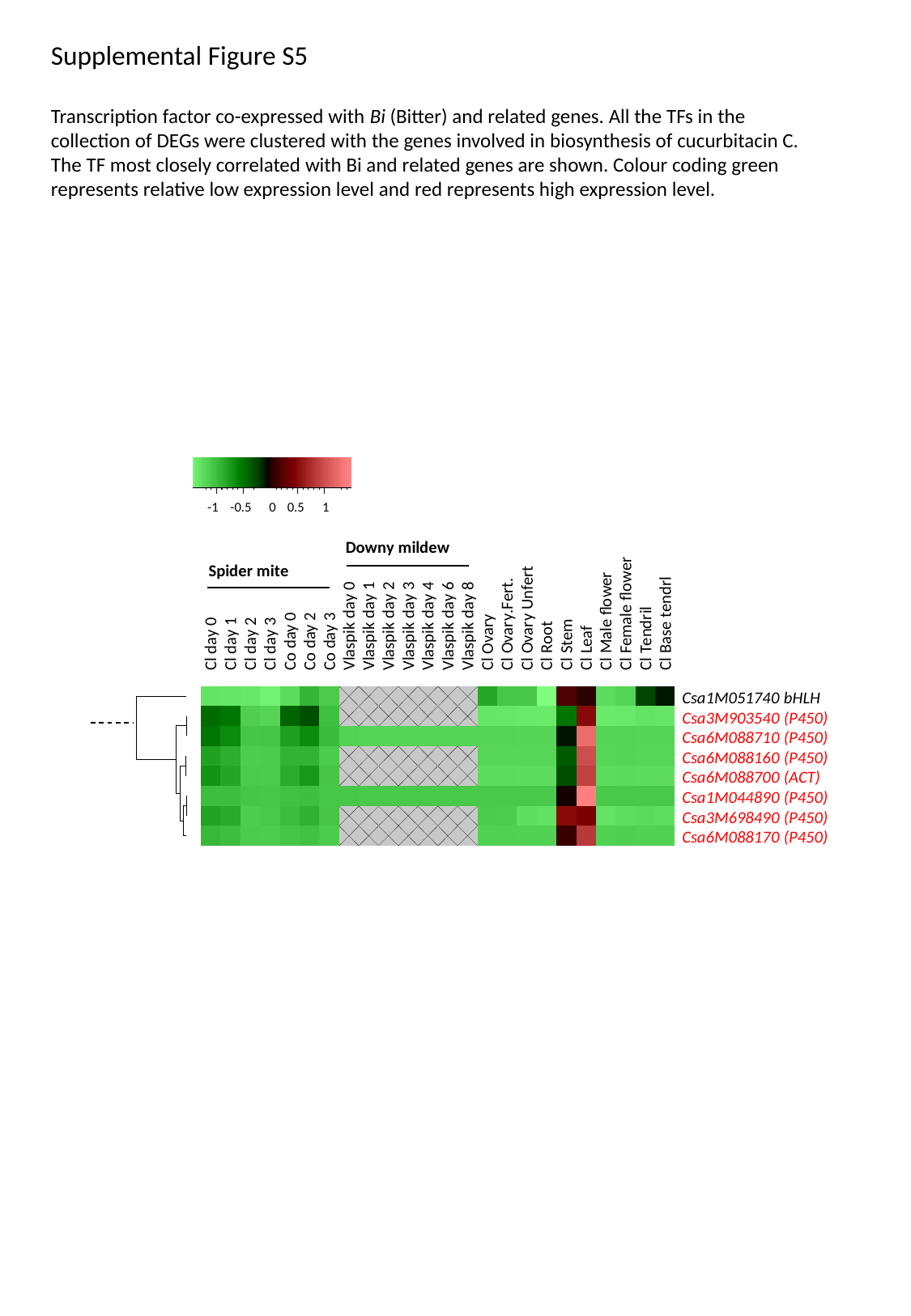

Supplemental Figure S5
Transcription factor co-expressed with Bi (Bitter) and related genes. All the TFs in the collection of DEGs were clustered with the genes involved in biosynthesis of cucurbitacin C. The TF most closely correlated with Bi and related genes are shown. Colour coding green represents relative low expression level and red represents high expression level.
